# Hemorrhoids

**Published:** 1858-01

**Authors:** 


*Hemorrhoids.*—Dr. Van Holsbeek recommends the following formula as of remarkable efficacy in the various forms of hemorrhoids, provided that these are uncomplicated: ℞ Sulphuri loti, sacchari canarini, of each ℥j; ext. strychn. nux. vom. gr. vj. To be mixed with a sufficient quantity of tragacanth so as to form twenty-four lozenges. Of these two are to be taken the first day, the number being increased by one every day, until six are taken daily. The patient is then to keep at that number during four days, when he is to diminish it gradually until only two are again taken daily. If a radical cure is not by this time effected, he must follow the same course again. The amendment is, however, usually so rapid that the treatment at farthest lasts a week.—*Presse Med. Belge—Ib.*

